# Prevalence and predictive factors of depressive and anxious symptoms among healthcare professionals at Farhat Hached University Hospital in Sousse during COVID-19

**DOI:** 10.1192/j.eurpsy.2024.1069

**Published:** 2024-08-27

**Authors:** N. Belhadj Chabbeh, Z. Athimni, S. Chatti, F. Chelly, M. Bouhoula, N. Ben Arbia, A. Chouchane, A. Aloui, I. Kacem, M. Maoua, A. Brahem, H. Kalboussi, O. El Maalel

**Affiliations:** ^1^Occupational Medicine and Professional Pathologies Department, Sahloul Teaching Hospital, Tunisia; ^2^Occupational Medicine and Professional Pathologies Department, Farhat Hached Teaching Hospital, Avenue Ibn El Jazzar, Sousse, 4000, Tunisia, Sousse, Tunisia

## Abstract

**Introduction:**

The COVID-19 pandemic was originally of a magnitude exceeding that of previous epidemics, placing a heavy burden on healthcare systems in general and healthcare professionals in particular.

**Objectives:**

To assess the prevalence of depressive and anxiety symptoms among healthcare professionals at Farhat Hached Hospital in Sousse during the COVID-19 pandemic and to identify associated risk factors.

**Methods:**

This is a descriptive cross-sectional study conducted among healthcare professionals practicing in the Farhat Hached Hospital of Sousse, which took place during the period extending between August and November 2021. The 9-item Patient Health Questionnaire (PHQ-9), the 7-item Generalized Anxiety Disorder (GAD-7), were used to assess depression and anxiety respectively.

**Results:**

Our study included 326 healthcare professionals from the Farhat Hached University Hospital. The mean age of our population was 36.38 ±10.19 years, with a clear female predominance of 81.3%. The prevalences of depression and anxiety were 46% and 35.3% respectively. Female gender and being a paramedical staff were risk factors for depressive and anxiety symptoms. On the other hand, professional seniority of over 5 years was statistically significantly associated with anxiety. Sporting activity was inversely associated with depressive and anxiety symptoms.

The multi-variate analytical study revealed that the practice of leisure activities other than sport was a protective factor against the occurrence of depressive symptomatology in healthcare professionals, while current smoking and the practice of a sporting activity were inversely associated with the occurrence of anxious symptomatology.

**Conclusions:**

These findings underline the need for specific prevention strategies to reduce these symptoms and help healthcare professionals maintain their mental health. This will help to guarantee the quality and efficiency of work in the medical environment, to better control the pandemic.

**Disclosure of Interest:**

None Declared

